# 4582 NICOTINAMIDE ADENINE DINUCLEOTIDE (NAD) DEPLETION MUST BE SEVERE TO INDUCE CARDIAC DYSFUNCTION AND EVENTUAL FAILURE

**DOI:** 10.1017/cts.2020.83

**Published:** 2020-07-29

**Authors:** Timothy Luongo, Lin Wang, Karthikeyani Chellappa, Melanie R. McReynolds, Daniel P. Kelly, Joshua D. Rabinowitz, Joseph A. Baur

**Affiliations:** 1 University of Pennsylvania

## Abstract

OBJECTIVES/GOALS: Nicotinamide adenine dinucleotide (NAD) plays essential roles in energy metabolism and cell signaling pathways. NAD functions as a coenzyme by accepting electrons during glycolysis and the TCA cycle and subsequently donates them to complex I of the electron transport chain providing the driving force for ATP production. NAD also acts as a co-substrate for several classes of enzymes, including sirtuin deacetylases. Both NAD and the enzyme that is rate limiting for synthesis, Nicotinamide phosphoribosyltransferase (Nampt), are depleted in the failing heart, concurrent with hyperacetylation and mitochondrial dysfunction. Moreover, treatment with NAD precursors reduced cardiac injury in several heart failure models. However, NAD precursors may have systemic effects, and it remains unproven whether depletion of myocardial NAD is causative or merely correlative for the onset and progression of heart failure. METHODS/STUDY POPULATION: To test this, we generated a cardiac-specific tamoxifen-inducible (αMHC-MerCreMer) model for deletion of Nampt (Nampt cKO) in cardiomyocytes. Adult mice were administered tamoxifen for 5 days leading to deletion of *Nampt*, resulting in a 72% reduction in myocardial NAD after two-weeks. RESULTS/ANTICIPATED RESULTS: Echocardiography revealed that Nampt cKO mice displayed a significant reduction in left ventricular (LV) contractility as well as cardiac hypertrophy. Despite the further loss of NAD, the majority of animals survived to 8 weeks of age before experiencing sudden deaths resulting in significant mortality over the next several weeks. Remarkably, we observed only a slight increase in acetylation of mitochondrial proteins, and cardiac mitochondria isolated from Nampt-null mice even at 8 weeks displayed a normal or higher oxygen consumption rate. We found that mitochondrial NAD levels were preferentially maintained and depleted at a slower rate compared to those in bulk tissue. DISCUSSION/SIGNIFICANCE OF IMPACT: While mild depletion of cardiac NAD has been reported in heart failure, our data indicate that the heart can adapt to much more severe loss of NAD prior to the loss of viability.

